# Fertility awareness-based mobile application for contraception

**DOI:** 10.3109/13625187.2016.1154143

**Published:** 2016-03-22

**Authors:** Elina Berglund Scherwitzl, Kristina Gemzell Danielsson, Jonas A. Sellberg, Raoul Scherwitzl

**Affiliations:** ^a^ NaturalCycles Nordic AB, Stockholm, Sweden; ^b^ Division of Obstetrics and Gynecology, Karolinska Institutet and University Hospital, Stockholm, Sweden

**Keywords:** Basal body temperature method, birth control, menstrual cycle, natural family planning methods, ovulation detection, pregnancy rate, retrospective study

## Abstract

**Objectives:** The aim of the study was to retrospectively evaluate the effectiveness of a fertility awareness-based method supported by a mobile-based application to prevent unwanted pregnancies as a method of natural birth control. **Methods:** In a retrospective analysis, the application’s efficiency as a contraceptive method was examined on data from 4054 women who used the application as contraception for a total of 2085 woman-years. **Results:** The number of identified unplanned pregnancies was 143 during 2053 woman-years, giving a Pearl Index of 7.0 for typical use. Ten of the pregnancies were due to the application falsely attributing a safe day within the fertile window, producing a perfect-use Pearl Index of 0.5. Calculating the cumulative pregnancy probability by life-table analysis resulted in a pregnancy rate of 7.5% per year (95% confidence interval 5.9%, 9.1% per year). **Conclusions:** The application appears to improve the effectiveness of fertility awareness-based methods and can be used to prevent pregnancies if couples consistently protect themselves on fertile days.

## Chinese abstract

目的：本研究的目的是回顾性评价一个建立在生育意识基础上的移动应用程序作为一种自然的生育控制方法，防止不必要的妊娠的有效性。方法：在回顾性分析中，通过4054名妇女对该应用程序共2085妇女年使用的资料，分析该应用程序作为一种避孕方法的有效性。结果：在2053妇女年的使用中，计划外妊娠是143次，典型使用的珀儿指数是7.0。10次妊娠是由于应用程序错误地认为受孕期是安全期，产生完美的珀儿指数为0.5。通过寿命表分析计算的累积妊娠概率为每年7.5%（95%的置信区间为每年5.9%-9.1%）。结论：应用程序改善以生育意识为基础的避孕方法的有效性，如果夫妻在排卵期坚持保护自己，它可以用来防止妊娠。

## Introduction

Fertility monitors have become increasingly popular in recent years as a tool to prevent pregnancies, due to rising interest among women to abstain from hormonal contraception.[1–3] It has been shown that the effectiveness of such devices when correctly used can be high,[3–5] competing with hormonal methods of birth control. These devices tell women about their current fertility, enabling them to make informed decisions with regard to family planning. The disadvantages of current fertility monitors are, however, that they are often expensive, difficult to use, make use of basic mathematical algorithms and, in some cases, lack clinical research.[4,6–9]

In this paper, we present a mobile-based application (known as Natural Cycles) that is used in combination with a conventional basal thermometer to identify ovulation and, hence, the fertile window. The application aims to be both safe and easy to use – two essential attributes that make a birth control method effective.[10] The basic features and functionalities of the application are described and the contraceptive effectiveness is assessed through a retrospective observational study of 4054 women using the application for 2085 woman-years.

## Methods

### Digital fertility monitor

The mobile application requires the input of basal body temperature recordings and the date of menstruation. Luteinising hormone (LH) test results are optional entry points. The required basal thermometer and the optional LH tests are acquired separately from the application. The users enter their fertility-related data into a device such as a smartphone, tablet or laptop computer.

The underlying technology is a statistical algorithm [11] that returns a red (unsafe) or a green (safe) day to the user depending on whether she is considered to be at risk of getting pregnant. The algorithm computes the following parameters and their uncertainties: ovulation day, luteal phase, follicular phase and cycle length, and the average temperatures of the different phases. The algorithm assigns green days in a conservative manner. Subsequently, the number of red days per cycle is generally greater than the empirical value of six days.[12,13] For example, for a woman who has a regular cycle with an average length of 28 days and ovulates regularly on day 14, the application would show red days from day 6 to day 16. It has been demonstrated in a previous study [11] that the algorithm can identify the ovulation day with high precision and that the probability of a green day being falsely attributed within the fertile window, surrounding the ovulation day, is 0.05%. Since ovulation day is accurately detected, various parameters can be tracked, such as the length and variation of the follicular and luteal phases of the cycle and the rate of anovulatory cycles.

The algorithm learns from previously recorded cycles from the same woman and can provide predictions of her fertility status and upcoming ovulation, LH and menstruation days. The current and predicted fertility status of the individual user is visualised through a status bar, a calendar view and a temperature graph. Statistics of the characteristics of recorded cycles are also displayed, which can be shared with physicians. Messages based on the user’s specific data are sent out to further advise and motivate the user to take measurements as frequently as possible and to repeatedly warn her to use protection on fertile days.

In addition to menstruation, basal body temperature and LH test results, it is also possible for the user to enter information concerning sexual activity and personal notes. If there is a possibility that the user has become pregnant, she is encouraged to enter a pregnancy test result to either confirm or contradict the pregnancy. The possibility of pregnancy is detected by scrutinising the data to search for a combination of: (1) delayed menstruation, identified by comparing the time elapsed since the last ovulation with the user’s normal luteal phase duration; and (2) consistently high temperature levels, as progesterone increases rather than decreases in the body if a fertilised egg has been implanted in the uterus.[14]

All the data are stored in the company’s database and can be exported for analysis by third, independent parties. In this study, the researchers from the company performed the analyses and communicated with the users, e.g., sent out the final questionnaire via email.

### Study design

This retrospective analysis observed fertile women aged 18–45 years from Sweden. The women had registered to use the application for the purpose of preventing pregnancies and were included in the study based on registration from 1 August 2014 to 31 March 2015. Women were recruited using conventional end-consumer marketing techniques (e.g., public relations, online and offline advertisements with taglines such as ‘prevent pregnancies naturally’). They purchased subscription to the application and the basal thermometer for €50. Every user who registered for the application agreed to share her data anonymously for subsequent research and clinical studies performed by the company or external researchers. Thus, every registered user effectively became a participant in the study if the following inclusion criteria were met:

- The participant had to have access to the application for at least three months during the study period.
- The participant had to enter data for at least 20 days in total. Each daily data point can be any kind of combination of the possible entries (menstruation, temperature, LH test results, sexual activity or a personal note) for a specific date.
- The participant had to be older than 18 years and not planning a pregnancy during the study period.

The only applied exclusion criterion was related to women with a medical condition in whom becoming pregnant would be dangerous to them or their fetus, as outlined in the instructions for use of the application. Women with irregular cycles were not excluded. No research centres, clinics or health care professionals were involved in recruitment or throughout the study, which was performed entirely digitally.

Because of the loose inclusion/exclusion criteria as well as the recruitment procedure, the study participants represented a general population of women who were susceptible to the marketing of the company, i.e., they had an interest in natural contraception.

At registration, users were required to answer questions related to their individual cycle, previous contraception, date of birth, height and weight. The first registration dating from 1 August 2014 marked the beginning of the study. Participants were subsequently recruited from 1 August 2014 to 31 March 2015. They entered data until the study ended on 31 August 2015 or until they dropped out due to pregnancy or method discontinuation. Pregnancy was initially detected via the user’s data, as described above, and secondly via an online questionnaire.

Approximately three weeks prior to the end of the study, the participants were asked to answer an additional optional questionnaire, sent via email. The survey contained the questions and answers presented in Table 1, and all questions were optional, except the question whether she had become pregnant (Q6), which was mandatory to answer to complete the survey. The answers were collected no later than 31 August 2015. Of 4054 women participating in the study, 1233 women completed the survey.

**Table 1.** Questions and answers used in this study from the survey that was sent to participants on 5 August 2015. All questions were optional except Q6, which was mandatory to answer to complete the survey; 1233 women contributed to the survey and 1186 women answered the mandatory question.

Question no.	Question	Possible answers
Q1	I use Natural Cycles as a method to:	Prevent pregnancy
		Track my cycles (premenstrual symptoms, period, ovulation, etc.)
		Get to know my body better
		All of the above
		To get pregnant
		Other (please specify)
Q2	Do you check Natural Cycles before having intercourse?	Yes
		No
		Sometimes
Q3	Do you use protection on red days?	Yes
		No
		Sometimes
Q4	Which method do you use on red days?	Condom
		Withdrawal
		Diaphragm
		No contraception
		Abstinence
		Other (please specify)
Q5	Do you have children?	No
		I have one child
		I have two children
		I have three children
		I have four or more children
Q6	Did you get pregnant while using Natural Cycles?	Yes
		No
Q7	If yes on Q6, was this on a:	Red day
		Green day
		Not sure exactly when it happened
Q8	Which method of birth control did you use prior to Natural Cycles?	The pill
		Long-acting reversible contraceptives
		The implant (P-stav)
		Condoms
		Abstinence
		Withdrawal
		Fertility awareness methods
		Other (please specify)
Q9	Are you happier since switching to Natural Cycles?	Yes
		No
Q10	Would you recommend Natural Cycles to a friend?	Yes
		No
		Other (please specify)

The study protocol was reviewed and approved by the regional ethics committee (EPN, Stockholm, diary number 2015/1363-31/4). The retrospective study format was chosen in order to assess for the first time the contraceptive effectiveness of fertility awareness-based methods supported by a mobile application. The available dataset in the company’s database allows for a cost-effective analysis, which provides insight for the design of future prospective and randomised trials. It can be of interest to study the results on contraceptive effectiveness in relation to the study design.

### Pearl Index

**Figure 1.**
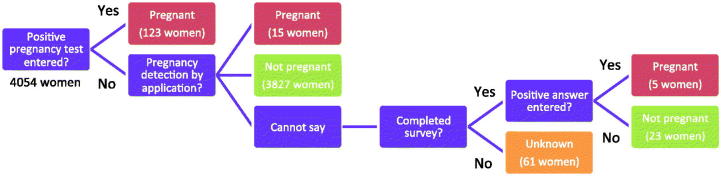
Flow chart describing how pregnancies are detected in the study (4054 women).

To determine the effectiveness of the application as a contraceptive method, the most crucial component of the analysis was to determine the number of pregnancies among the study participants. This information can be used to calculate Pearl Indexes [15] for perfect and typical use. Pregnancies were identified directly from the data of a user entering a positive pregnancy test or by the algorithm detecting a possible pregnancy as described above. The answers from the questionnaire were an additional way of determining pregnancies (Figure 1). In order to estimate the most conservative Pearl Index, all users considered potentially pregnant by the algorithm were classified as pregnant in this study even if they failed to confirm with a pregnancy test as requested. If it was not possible to detect a pregnancy with any of the three methods, or to exclude the occurrence of pregnancy from the user’s data, we classified this specific case as unknown.

If a green day had been given within the fertile window in a cycle where a woman had become pregnant, we considered it a method failure, irrespective of whether she had logged intercourse and even if she had indicated unprotected intercourse on red days closer to ovulation. In this manner, the most conservative perfect-use Pearl Index was determined independently on logged sexual behaviour.

In addition to determining the Pearl Index, life-table analysis was used to calculate the cumulative probability of pregnancy on a cycle basis using the Kaplan–Meier estimator.[16,17]

## Results

The application was tested by 4054 participants, and a total of 483,221 daily data entries were analysed. The participants on average added data on 63% of the days, of which 88% were temperature data, 4% were LH test results and 8% intercourse information.

The age, parity and body mass index (BMI) distribution of the participants of the study are shown in Table 2. Table 3 shows contraceptive usage prior to use of the application, as well as contraceptive usage for days when the application returned red (fertile) days. In the daily data entries, the users had the option to log information about their sexual activity. Since it was not mandatory, users only spontaneously logged this information. Unprotected and protected intercourse was logged on 4.1% and 2.0% of the days, respectively, when daily data were entered (e.g., in addition to temperature). Participants also logged that they did not have sexual intercourse on 2.3% of the days when they entered daily data. When asked whether protection was used on red days, 67% of women (803 out of 1193) answered ‘yes’, 20% (237 out of 1193) answered ‘sometimes’ and 13% (153 out of 1193) answered ‘no’ (40 out of 1233 women skipped the question).

**Table 2.** Age and BMI distributions among all participants in the study not listed as unknown in Figure 1 (3993 women). The distribution of number of children for the subset of women who answered the questionnaire is also presented.

Variable	Number of women	Percentage of women
Age (years)		
<20	54	1
20–24	1263	32
25–29	1729	43
30–34	672	17
35–39	205	5
≥40	70	2
BMI (kg/m^2^)		
<20	709	18
20–25	2397	60
≥25	887	22
Parity		
No children	935	79
1	130	11
2	93	8
3	16	1
≥4	11	1

**Table 3.** Previous contraceptive method prior to the study, as well as chosen contraceptive method for red (fertile) days given by the application. The data were provided by 1233 women who answered the questionnaire. Women were able to select multiple choices or skip the question(s) completely.

Contraception	Number of women	Percentage of women
Contraception usage prior to using Natural Cycles		
Hormonal contraceptive pill	748	65
Condom	146	13
Hormonal implant	66	6
Intrauterine device	25	2
Withdrawal	38	3
Abstinence	10	1
Fertility awareness methods	15	1
Other	109	9
Contraception usage during red (fertile) days		
Condom	871	74
Withdrawal	379	32
Abstinence	162	14
No protection	82	7
Diaphragm	7	1
Other	30	3

Among the 4054 women using the application as a contraceptive method during a total of 2085 woman-years, 143 pregnancies were identified: 123 were detected through positive pregnancy test entries in the application; 15 of the women were considered pregnant by the application’s pregnancy detector; and five additional pregnancies were found through the survey. Thirty-four percent of the women (1397 out of 4054) discontinued using the application prior to the end of the study. Among these dropout cases, the pregnancy status of 61 participants was classified as unknown. Excluding these women from the study gives a Pearl Index of 7.0 for typical use. To estimate the most conservative upper limit of the Pearl Index, all unknown cases were considered as pregnant, yielding a value of 9.8. Note that 1.8% of women (21 out of 1194) answering this question in the survey declared that the purpose of using the device was to plan a pregnancy, showing that they had altered their intended use during the course of the study period. The subset of pregnancies that could possibly have occurred during intercourse on a green day amounted to 10 out of 3993 women, yielding a method failure for perfect use of 0.5 according to the Pearl Index. Table 4 summarises the estimated Pearl Index values for the different scenarios.

**Table 4.** Pregnancy classification and contraceptive efficacy calculated according to the Pearl Index based on woman-years.

Sample	Participants (*n*)	Pregnancies (*n*)	Woman-years (*n*)	Pearl Index
Typical use	3993	143	2053	7.0
Typical use, upper limit	4054	204	2085	9.8
Perfect use	3993	10	2053	0.5

**Figure 2.**
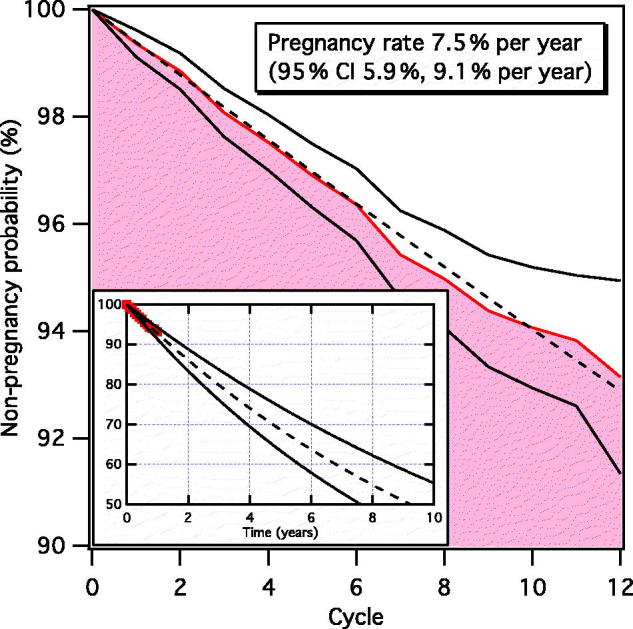
The probability of not becoming pregnant over time, measured in ordinal cycle number. The filled red area is based on experimental data from Table 5 and black solid lines are the corresponding 95% CIs. The black dashed line is the fitted probability of not becoming pregnant, from which the pregnancy rate is calculated. The inset shows the evolution of the non-pregnancy probability over several years predicted by the fitted pregnancy rate (black dashed line). Experimental data are here shown as a red line, whereas black solid lines correspond to the fitted pregnancy rates of the 95% CI.

Life-table analysis presenting the number of women exposed to the risk of becoming pregnant and the number of cumulative pregnancies by cycle is shown in Table 5. The cumulative probability of pregnancy and its 95% confidence interval (CI) were calculated according to the Kaplan–Meier estimator [16,17] and resulted in a pregnancy probability after 12 cycles of 6.85% (95% CI 5.06%, 8.65%). Regression analysis based on the life-table data is shown in Figure 2 and resulted in a fitted pregnancy rate of 7.5% per year (95% CI 5.9%, 9.1% per year), which is slightly higher than the estimated typical-use Pearl Index of 7.0. The inset of Figure 2 shows the extrapolated non-pregnancy probability predicted by the fitted pregnancy rate. Over a time span of 10 years, we estimate that 52.8% (95% CI: 44.7%, 59.8%) of the women will become pregnant.

**Table 5.** Life-table analysis presenting the number of women exposed to the risk of becoming pregnant by cycle, the number of cumulative pregnancies, the cumulative pregnancy probability and its 95% CI. The cumulative pregnancy probability is calculated on a cycle basis, where cycle 1 corresponds to the first cycle for which the ovulation day was detected and cycle 12 implies that the women were exposed to at least 11 full cycles after cycle 1. 60 out of 3993 women dropped out prior to detection of their first ovulation day and are therefore censored before cycle 1.

Ordinal cycle number	Women exposed	Cumulative pregnancies	Cumulative pregnancy probability (%)	CI, lower bound (%)	CI, upper bound (%)
1	3933	25	0.64	0.39	0.88
2	3656	44	1.15	0.81	1.49
3	3324	70	1.93	1.48	2.37
4	3020	87	2.48	1.96	2.99
5	2674	104	3.10	2.51	3.69
6	1981	115	3.64	2.97	4.30
7	1443	129	4.57	3.75	5.39
8	1058	134	5.02	4.11	5.93
9	798	139	5.62	4.57	6.66
10	601	141	5.93	4.81	7.06
11	389	142	6.17	4.95	7.39
12	138	143	6.85	5.06	8.65

We found that the lengths of the average cycle and luteal phase, and their variation, were 29.9 ± 4.1 days and 12.7 ± 1.4 days (1 standard deviation [SD]), respectively. The rate of anovulatory cycles was also monitored and found to be 5.1%.

The fraction of green days returned to the user is an important measure of usability and depends on several factors such as cycle regularity, length of the luteal phase, quality of the temperature data, whether LH is measured, and whether the user has recently used hormonal contraception. A user with a regular cycle and normal temperature fluctuations can expect 58 ± 12% green days (1 SD) after the initial learning period, assuming that she regularly enters temperature data. In general, cycles with a positive LH result have 5% more green days than cycles with only basal body temperature information. As the application learns from previous data, the number of green days increases per recorded cycle. We found that the application yields 41% green days on average in the first recorded cycle. Women with less than 50% green days had a 41% higher likelihood of discontinuing the method than those with more than 50% green days.

In the survey performed at the end of the study, several questions were included concerning the level of satisfaction with the application. When asked whether she was happier since switching to using the application as a contraceptive method, 83% of women (955 out of 1156) answered ‘yes’. In response to the question whether she would recommend the method to a friend, 88% of women (1038 out of 1178) answered ‘yes’ and only 6% (75 out of 1178) answered ‘no’ (the remaining users answered ‘other’, with the addition of a comment). Interestingly, 45% of women (64 out of 143) who became pregnant continued to use the application after they had entered a positive pregnancy test, either for the purpose of tracking their pregnancy or as contraception after having had an abortion or miscarriage.

**Figure 3.**
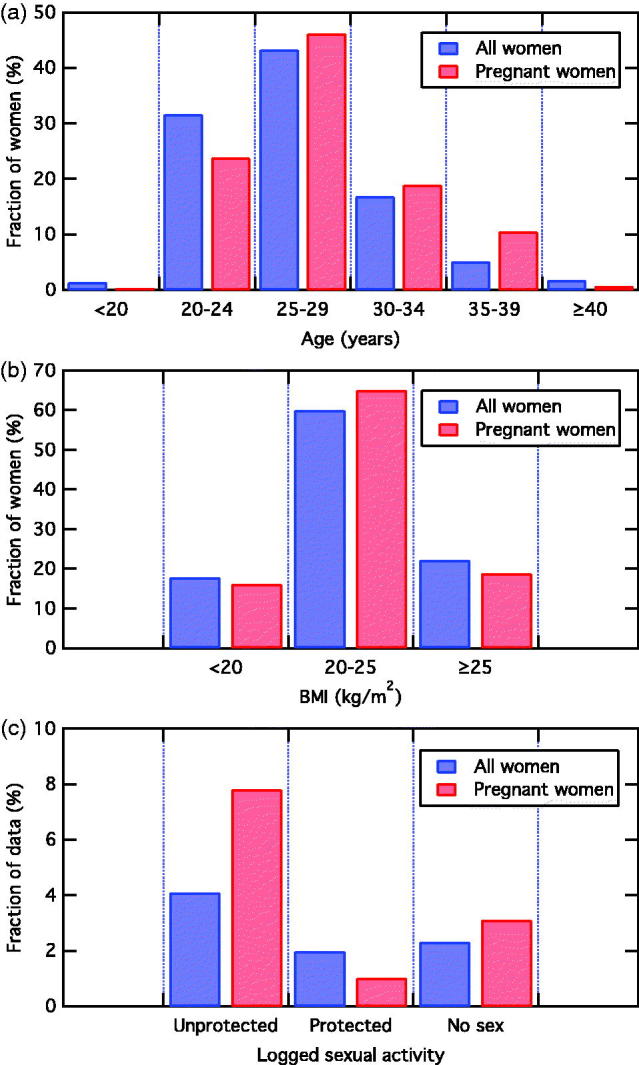
Comparison of age (a) and BMI (b) distribution of all participants (blue) listed in Table 2 with that of only pregnant women (red). Both distributions are normalised to the total number of women who make up each distribution (3993 women and 143 pregnant women, respectively). (c) Comparison of logged sexual activity for all women (blue) and pregnant women (red), in terms of unprotected intercourse, protected intercourse and no intercourse, normalised to the total number of data entries for each sample group.

The characteristics of the 143 women who became pregnant while using the application were compared with the total sample of 4054 women in the study. The average age of the women who became pregnant was higher than that of all participants (Figure 3a), while the BMI distribution was determined to be equivalent within statistical uncertainty (Figure 3b). In addition, the fraction of anovulatory cycles and the fraction of women previously using hormonal contraception are analogous. A large discrepancy was, however, found in the rate of unprotected and protected intercourse as seen in the daily data entries. The sample of pregnant women had 91% more unprotected intercourse and 49% less protected intercourse than the average woman in the study (Figure 3c). We also saw that the users in the pregnant sample had overall 45% more intercourse in general.

Investigating the fertile window in the 143 cycles where a pregnancy occurred, 51% of the women had logged unprotected intercourse during the fertile window and only 3% had logged protected intercourse. These findings thus suggest that the majority of the pregnancies were due to lack of use of protection rather than failure of use of a barrier method. The fraction of green days was compared between the pregnant women and the total sample. Pregnant women were found to have one additional red day per cycle on average, which is not statistically significant.

## Discussion

### Findings and interpretation

The findings on the effectiveness of fertility awareness-based methods vary greatly in the literature depending on the fertility indicators used (menstruation, temperature, cervical mucus, or a combination), the social settings (industrialised or developing countries) and the study design itself. While a perfect-use Pearl Index has been reported to be low (0.4-4.0) for methods involving at least temperature or cervical mucus, the typical-use Pearl Index ranges from 1.8 to 24.0.[5,18–21] The World Health Organization (WHO) performed a prospective study in five countries using the ovulation method, which only investigates cervical mucus, and found an average typical-use Pearl Index of 20.4 in the effectiveness phase, after correction.[20] In a prospective study in Germany, the sympto-thermal method, involving both temperature and cervical mucus, revealed a very low typical-use rate of 1.8.[5] Studies using computer thermometers, which are similar to the current application, showed a typical-use rate of 3.8.[4]

The effectiveness of Natural Cycles as a contraceptive method depends on the accuracy of the algorithm as well as the behaviour of the user. The algorithm has been demonstrated to have a low failure rate, with a 0.05% probability of a green day being falsely attributed to the fertile window.[11] The estimated perfect-use Pearl Index of 0.5 confirms the high safety of the green days returned by the application. Considering the number of green days given by the application during the course of this study, as well as the probability of conception according to Wilcox *et al.*,[12] one would expect 22 pregnancies if users only had unprotected intercourse on green days within the fertile window. The finding of 10 pregnancies due to method failure is thus compatible with these results if users had unprotected intercourse on approximately every other such green day. The perfect-use rate is consistent with those reported on similar devices and for the sympto-thermal method.[4,5]

In this paper, user behaviour is also evaluated by determining a typical-use Pearl Index and Kaplan–Meier pregnancy rate, which include pregnancies occurring from intercourse on red (fertile) days. The resulting values of 7.0 and 7.5% per year, respectively, are significantly lower than the general typical-use Pearl Index of 24.0 during the first year for fertility awareness-based methods.[18,21] The main reasons for the failure were reported to be due to a conscious departure from abstaining or using protection on fertile days and the inaccurate interpretation of fertility indicators. A fertility monitor, such as that studied here, may help to reduce these factors, since an algorithm automates the analysis of the temperature data. According to the survey in the present study, 74% and 32% of the participants made use of the condom or withdrawal, respectively, during fertile days (Table 3). Both methods have similar perfect- and typical-use Pearl Index values of 2.0–4.0 and 18.0–22.0, respectively.[18,21] Users are reminded through the application that a pregnancy risk exists on a red day, which may increase compliance with the use of a condom or withdrawal. In addition, the participants in this study were recruited from Sweden, a high-resource country whose inhabitants may behave differently from participants in the WHO study in the Philippines, India, El Salvador, Ireland and New Zealand. Studies on German women have shown a typical-use Pearl Index for the sympto-thermal method of 1.8 [5] and for a computer thermometer of 3.8.[4] Both rates are lower than in this study. The study of the computer thermometer is perhaps most similar to this application, for which the difference in failure rate may be explained by the study design. The retrospective German study [4] was entirely based on surveys sent by mail to women who had previously purchased the device, which could lead to a downward selection bias. The study of the sympto-thermal method [5] showed that the commitment of the participants to abstain or use protection on fertile days was high and that risk-taking couples (who had unprotected intercourse) had an unintended pregnancy rate of 7.5%, which is comparable to the result in this study. The fact that two fertility indicators are used rather than one may also lead to improved method failure rates, whereas effective teaching and high selection bias on recruitment may lead to low user failure rates.

It is clear that the users who became pregnant in this study were more likely to expose themselves to risk by not using protection on fertile days. It is thus of highest importance to educate the user on the risks involved in having unprotected intercourse on red days. In addition, the finding that users who became pregnant were older indicates that the pregnancy risk was of less importance.

Note that the safety of the algorithm is set mathematically and was regulated to achieve a balance between high safety of green days (low perfect-use Pearl Index) and a sufficient number of green days both to keep users satisfied with the method and to keep using protection on all red days (low typical-use Pearl Index). The fraction of green days returned to the user is similar to that of other computer thermometers and did not seem to affect pregnancy rates.[4] A low fraction of green days (<50%) does, however, lead to more dropouts.

The survey performed at the end of the study showed that a high level of satisfaction with the application was achieved. Conclusions from the survey must be critically assessed, however, since only 30% responded to the survey.

### Strengths and weaknesses of the study

This was a retrospective study with the disadvantages inherent to this type of study. However, by analysing the data that the users entered directly into the application on a daily basis, the pregnancy status of 3965 out of 4054 women could be detected directly by the application. Thus, the recall bias of the study was lower in comparison with solely basing the study on a survey performed at the end of a study period.[4] In addition, since pregnancy status could be determined for 98.5% of all participants (3993 out of 4054 women), the retention bias was low and the follow-up rate high. Since participants purchased and used the application as they would in real life, without any interaction with physicians, which is otherwise typical of such clinical studies, the results of this study may reflect a more accurate picture of how women use the application.

A disadvantage of this study is its shortness. As the study ended less than 5 months after recruiting the last participants, the average number of cycles per user was rather low (6.3 cycles per user). Taking the dropout rate of 34% into account, the expected one-year discontinuation rate of all participants is estimated to be 56%, which is comparable to the general one-year discontinuation rate for fertility awareness-based methods, but worse than for oral contraceptive pills or long-acting reversible contraceptives.[18,21] No incentives were given to the study participants and the contraceptive method was not free of charge, as is usually the case in prospective controlled trials. These factors could have had a negative impact on the continuation rate, which will be re-evaluated in a randomised, prospective study.

The information on sexual behaviour is limited, since it was not mandatory for participants to log whether and when they had protected/unprotected intercourse. Information about intercourse was logged on 8% of the days with daily data entries. Consequently, a calculation of perfect/imperfect use according to Trussell and Grummer-Strawn [20] could not be made. Instead of determining all cycles (and corresponding pregnancies) in which the method was used perfectly by the user, the perfect-use failure rate was determined by retrospectively analysing all cycles in which the algorithm failed (e.g., falsely attributing a green day within the fertile window). To obtain the most conservative estimate, cycles in which a pregnancy occurred when a green day had been falsely attributed were counted as method failure, even when the user had not followed protocol. The relative analysis on sexual behaviour between pregnant and non-pregnant participants is uncertain due to the small data subset, which makes any conclusion speculative, but the findings are consistent with the fact that ‘risk-takers’ are more likely to get pregnant.

Lastly, we note that the study population only contained women who had already chosen to use the application, which might have led to a high selection bias compared with the average population. Also, since 92% of the women were aged between 20 and 35 years, the results found in this study are not relevant to other age groups (e.g., teenagers).

### Differences in results and conclusions

The conclusions are based on the results from the retrospective analysis, but also include a comparison with other fertility awareness-based methods [18,21] discussed above.

### Relevance of the findings: implications for clinicians and policy-makers

The findings give the first indications of the effectiveness of the application when it is used to prevent pregnancies. We note that to be able to directly compare its efficacy with that of other contraceptive methods which also depend on high user compliance, such as the combined oral contraceptive pill and other forms of fertility awareness-based methods, further studies are needed that randomly assign the contraceptive method to the participant to avoid selection bias.

### Unanswered questions and future research

Many of the limitations discussed above could be improved by performing further studies with a longer time span and of a prospective nature. The platform renders it possible to perform clinical research on a large number of women and analyse pregnancy rates as a function of geography, age, BMI, data activity, educational level, and other potentially interesting factors. Furthermore, it would be interesting to perform a randomised, prospective clinical study that compares the efficacy of the application with that of the combined oral contraceptive pill. Such a study would determine the Pearl Index with much less selection bias.

## Conclusions

The mobile application appears to be an improvement on traditional fertility awareness-based methods and is comparable to existing computer thermometers. It can be an effective means to prevent pregnancies if couples are willing to abstain or protect themselves on fertile days. The algorithm removes the need for the user to perform any analysis of fertility data herself and thus reduces the probability of failure due to the human factor, which is verified by the low typical-use pregnancy rate observed in the study. The discontinuation rate remains high, similar to other fertility awareness-based methods. Furthermore, the platform presents an interesting approach to perform clinical research in reproductive health in a large set of women in a cost-effective manner.

The users’ feedback on the application as a contraceptive method was positive and indicated that most women were happier than with their previous contraception. Future prospective studies with a longer time span should compare the effectiveness and user experience of the application with other fertility awareness-based methods as well as with hormonal contraceptives.
